# Microstructure and mechanical properties of subchondral bone are negatively regulated by tramadol in osteoarthritis in mice

**DOI:** 10.1042/BSR20194207

**Published:** 2020-09-04

**Authors:** Chen-Chen Ji, Bo Liu, Yi-Jie Shao, Ting Liang, Hua-Ye Jiang, Guang-Dong Chen, Zong-Ping Luo

**Affiliations:** 1 Department of Orthopedics, The First Affiliated Hospital of Soochow University, 899 Pinghai Road, Suzhou 215006, Jiangsu, P.R. China; 2 Orthopedic Institute, Soochow University, 708 Renmin Road, Suzhou 215006, Jiangsu, P.R. China

**Keywords:** Elastic modulus, Mechanical properties, Osteoarthritis, Subchondral bone, Tramadol

## Abstract

**Objectives:** In the treatment of osteoarthritis (OA), tramadol, a common weak opioid, has become popular due to its effectiveness in inhibition of pain. In the present study, we aimed to explore the effect of tramadol on subchondral bone, especially changes in the microstructure and mechanical properties.

**Methods:** A mouse model of OA was established in the present study by destabilization of the medial meniscus (DMM). A vehicle or drug was administered for 4 weeks. Specimens were harvested and analyzed radiologically and histologically using micro-computed tomography (micro-CT), scanning electron microscopy (SEM), atomic force microscopy (AFM) and histological staining to evaluate the knee joints of mice undergoing different forms of intervention.

**Results:** In the early stages of OA induced by DMM, the subchondral bone volume fraction in the OA group was significantly higher than in the sham+vehicle (sham+veh) group, while the volume in the treatment groups was lower than in the DMM+vehicle (DMM+veh) and sham+veh groups. In addition, the elastic moduli in the treatment groups clearly decreased compared with the DMM+veh and sham+veh groups. Observations of the subchondral bone surface by SEM indicated serious destruction, principally manifesting as a decrease in lacunae and more numerous and scattered cracks. Histological staining demonstrated that there was no difference in the degeneration of either the articular cartilage or synovial cells whether tramadol was used or not.

**Conclusion:** Although tramadol is effective in inhibiting pain in early OA, it negatively regulates the microstructure and mechanical properties of subchondral bone in joints.

## Introduction

Osteoarthritis (OA) is a degenerative, chronic disease of the joints that occurs globally, contributing to numerous physical complications and ranks fifth among all forms of disability and affects 10% of the world’s population [1]. Progression of OA is often accompanied by pain, motor dysfunction and even disability [2]. The number of OA patients in China currently exceeds 100 million [3]. The disease not only brings considerable suffering to patients and their families but is also a cause of huge loss of socioeconomic resources.

Based on the 2014 guidelines for the diagnosis and treatment of early OA, consensus has been reached that the use of weak opioid drugs such as tramadol provides the same relief from pain as non-steroidal anti-inflammatory drugs (NSAIDs) and so they have become the first-line drug of choice [4]. Tramadol is a synthetic analgesic with central activity, playing an analgesic role by inhibition of the reuptake of norepinephrine and 5-hydroxy tryptamine [5,6]. Compared with other opioids, it has a weaker interaction with μ-opioid receptors in addition to causing fewer adverse reactions such as respiratory inhibition or addiction [7,8]. According to the Cochrane meta-analysis conducted by Toupin et al. in 2019, tramadol causes a milder effect on the gastrointestinal tract and cardiovascular system than NSAIDs. Additionally, kidney problems that occurred by tramadol may appear more rarely than other pain relievers [9]. Tramadol or NSAIDs for symptomatic knee OA were recommended by American Academy of Orthopaedic Surgeons currently [10]. Along with NSAIDs, tramadol acts as a first-line therapy for patients with knee OA conditionally recommended by American College of Rheumatology guidelines from 2012 [11]. The application of tramadol in early OA is becoming more popular because of the intolerance to NSAIDs in patients with digestive system diseases or cardiovascular diseases. And another reason for the prevalence is the abuse of opioids prescriptions worldwide which was demonstrated by the increased rate of tramadol prescriptions for knee OA, from 5% in 2003 to 10% in 2009 [12]. To a certain extent, this treatment is effective in relieving suffering and improving the quality of life for OA patients [13–15].

There is uncertainty about whether the tramadol used to treat OA interferes with the function of osteoblasts through their effect on bone metabolism and inflammation, thereby inhibiting bone formation and decreasing bone mineral density [16–18]. If so, what are the changes that occur to the microstructure and mechanical properties of subchondral bone as a result? In the present study, OA in mice was induced by destabilization of the medial meniscus (DMM) then treated with tramadol, aiming to evaluate subchondral bone remodeling in the early stage of OA. Consequently, the purpose of the present study was to investigate the role of weak opioids in the process of subchondral bone degeneration and remodeling during the early stages of OA and to explore changes to the microstructure and mechanical properties of subchondral bone.

## Materials and methods

### Preparation of samples and grouping of experiments

Mice from the same batch of male C57BL/6J mice (8 weeks old) produced by JOINN Laboratories (License No. SCXK(Su)2018-0006, Suzhou, China), Inc. were used in the present study. The animal experiments were licensed by the Animal Protection and Use Committee of Soochow University. Following surgery, the mice were housed in an appropriate environment strictly in accordance with the standards of the specific pathogen-free animal laboratory. All mice were randomly divided into five groups each consisting of 15 mice: vehicle injection in the sham group, vehicle injection in the DMM group (DMM+veh) and different dosages of tramadol injection in DMM groups. Mice in the sham+vehicle (sham+veh) group received a sham operation on the right knee joint. The animal experiment was performed in the animal operation room of the Orthopedic Institute, Soochow University.

### Induction of OA models

OA was established in male 8-week-old C57BL/6 mice in the knee by DMM prior to analysis [19,20]. The mice were anesthetized with isoflurane by inhalation and the epidermis gently split after disinfection after which the fascia layer was peeled off. The right knee joint was exposed by shifting the extensor muscle. The medial meniscus ligament of the right knee was severed after entirely exposed, without transection of the patellar ligament. The incision was then sutured and the skin closed after reposition and immobilization of the anterior patellar ligament and the knee extensor. Mice with sham surgery got the same operation except for dissection of the medial meniscus ligament. The mice were provided with a balanced standard diet and water *ad libitum* with sufficient space for postoperative exercise.

### Experimental intervention and preservation of samples

The procurement and transportation of the medicines used in the present study (sevoflurane, tramadol and saline) were in compliance with the provisions of the China Food and Drug Administration. All injectable drugs were dissolved in normal saline. As in previous studies [21], three concentrations of tramadol (10, 20, 40 mg/kg) were injected into mice intraperitoneally three times per week for 4 weeks immediately after surgery, while the same volume of normal saline was injected into the abdominal cavity in the sham+veh and DMM+veh groups. Mice were killed by continuous CO_2_ inhalation and knee joints harvested 4 weeks after surgery. Specimens for micro-computed tomography (micro-CT) and histological analysis were preserved in formalin fixative and those destined for analysis by scanning electron microscopy (SEM) and atomic force microscopy (AFM) were stored in liquid nitrogen.

### Visual observation by SEM

The muscles and surrounding ligaments were carefully cut from the specimens after the mice were killed. The intact tibial plateau and a part of the tibial shaft were placed in a tube containing a mixed enzyme solution of type I and type II collagenase (4%) and then digested in a 37°C incubator for 1 week [22]. Following digestion of the articular cartilage of the tibial plateau, the subchondral bone of each sample was exposed. After washing with phosphate buffered saline (PBS), the samples were fixed in 4% glutaraldehyde then dehydrated through an increasing concentration gradient (70, 80 and 90%) of alcohol. Finally, the specimens were dried for 24 h in a cool and ventilated environment. The specimens were then fixed on to stubs with conductive glue and inspected by SEM after coating with gold ions.

### Analysis by micro-CT

After knee joints had been excised, they were fixed in 10% neutral formalin for 48 h, 70% ethanol for 48 h and then scanned using high-resolution micro-computed tomography (μCT; SkyScan, Belgium) [23]. Scanning was conducted using a 50 kV X-ray source voltage and 100 μA current to obtain images having a pixel size of 9 μm. The anterior subchondral bone of the medial tibial plateau (MTP) was analyzed by three-dimensional reconstruction and analysis software (CTan). All samples were analyzed using standardized parameters, including gray values of 0–0.075. BV/TV represents the ratio of bone volume to total volume and reflected the degree of bone mineralization. Average thickness of phalangeal trabeculae (Tb.Th), quantity of phalangeal trabeculae (Tb.N) and sparsity of phalangeal trabeculae (Tb.Sp) are parameters of average thickness, quantity and sparsity of phalangeal trabeculae, respectively.

### Measurement by AFM

To ensure that the mechanical properties of the subchondral bone were not affected, the surrounding muscles and ligaments were carefully removed without chemical treatment (for example, 10% neutral formalin fixation) after the mice were killed. Instead, the specimens were dehydrated by immersing in 20 and 30% sucrose solution for 12 h. Samples were then sectioned into 30-μm slices using a cryostat for analysis by AFM. First, the force constant k of the probe (Brooke Co., Germany) was found to be 40 N/m after thermal tune calibration. In addition, the radius of curvature of the AFM tip was measured to be 5 nm. The region of interest was scanned by AFM imaging. The software was programmed with loading and shrinkage rate constants, avoiding the effects of creep and inputting corrected parameters. In the present study, a Hertz model was used to calculate the compressive moduli of elasticity, the most commonly used model for biological tissue in AFM. The specific formula is as follows:

$$\text{F}=\frac{4}{3}\frac{E}{\left(1-\upsilon^{2}\right)}\sqrt{R}\delta^{3/2}$$

where F, E, v, R and δ represent pressure, Young’s modulus, Poisson’s ratio, indentation radius and indentation depth, respectively.

### Histological analysis of cartilage and synovitis

Joints were fixed in 10% formalin after removal, then decalcified in 10% EDTA for 2 weeks. The samples were then dehydrated in alcohol and embedded in paraffin, sliced into 6-μm-thick sections then stained with Hematoxylin and Eosin (HE) and Safranin O-Fast Green (SO/FG) (Sigma–Aldrich, St. Louis, Missouri, U.S.A.) for investigation of morphological changes in the tissues of the cartilage and subchondral bone of the joints. HE and SO/FG staining were used to evaluate degeneration of the cartilage of the tibial plateau. Articular cartilage can be categorized into hyaline cartilage (HC) and calcified cartilage (CC). In models where OA was induced by DMM, changes due to mechanical instability in the loading of the HC contributed to a reduction in its thickness. In the present study, the ratio of HC to CC was used as a measure of transformation of the articular cartilage [24]. Furthermore, continuity and glycogen loss from the surface of the cartilage were measured by SO/FG staining. The severity of degeneration was graded using the Osteoarthritis Research Society International (OARSI) scoring system [25]. The morphology and proliferation of synoviocytes and synovial lining cells in the joints were analyzed by HE staining. Semi-quantitative evaluation was performed to grade the severity of synovial pathological changes using a score of 0–6, in accordance with a study published in Osteoarthritis and Cartilage in 2011 by Lewis et al. [26].

### Statistical analysis

All comparisons between independent groups were calculated using Sigmaplot 14.0 using a one-way ANOVA and post-hoc Tukey’s test. Graphs were plotted using GraphPad Prism 8.0 software (San Diego, California, U.S.A.). Results were expressed as means ± the standard deviation (SD). Difference between groups were considered statistically significant for *P*-values less than 0.05.

## Results

### Observation by SEM revealed more severe bone destruction in the tramadol groups

**Figure 1:**
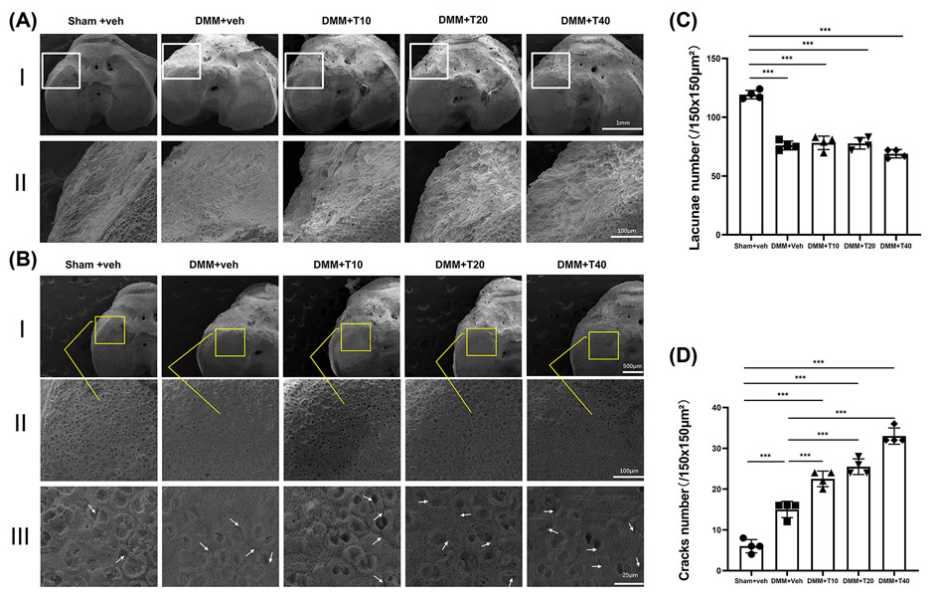
The bone destruction showed above as revealed in SEM (**A**) The superficial presentation anterolateral of tibia entocondyle. (**B**) The lacunae and cracks in load-bearing area of the knee joint. (**C,D**) Quantitative analysis of lacunae and cracks number in load-bearing area of the knee joints. Statistically significant differences are indicated by where ***P<0.001 between the indicated groups. The scale bars equaling different length were shown above.

Due to the high magnification inherent with SEM, the anterolateral aspect of the tibial entocondyle appeared flat, as observed in the sham+veh group. Sclerotin was distributed evenly. However, after OA induced by DMM, with or without intervention, superfluous osteogenesis had occurred, osteophytes on the lateral side, typical lesions of OA. Hyperplastic osteophytes exhibited a disordered texture, with a rough surface and deep depression under SEM (Figure 1A). The observations above were similar in the DMM+veh and treatment groups. The load-bearing region (LBR) of the knee joint is located on the anterior medial surface of the tibial plateau. According to Figure 1B, for further investigation on the microstructure changes of subchondral bone, we statistically analyzed the number of lacunae and cracks within the LBR of the subchondral bone. The lacunae and cracks on these surfaces represent the location of individual chondrocytes and microfracture lines respectively. At a magnification of 800×, the number of lacunae attached by chondrocytes on the surface of subchondral bone, in the sham+veh group was significantly higher than in the other four groups. However, there was no significant difference between DMM+veh group and treatment groups (Figure 1C). When magnified to 3000×, cracks were visible on the surface of the subchondral bone which were superficial and few in number in the sham+veh group. Conversely, deep and scattered cracks appeared in other groups whose number of cracks increased obviously with statistical significance (Figure 1D). Compared with DMM group, the number of cracks in different dosage groups increased significantly as well. Meanwhile, we found that the difference between treatment groups was too small to be statistically significant.

### Tramadol treatment contributed to an absolute loss of subchondral bone reflected by micro-CT

**Figure 2:**
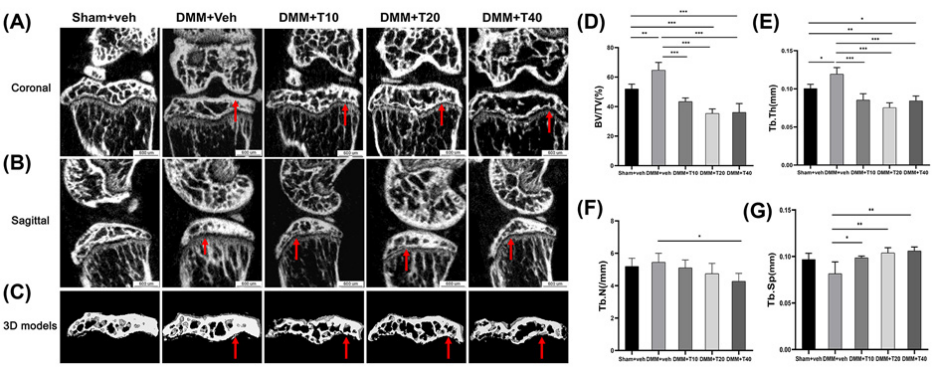
The treatment of tramadol had caused significant loss of subchondral bone mass of the knee joint as revealed in micro-CT (**A,B**) Coronal and sagittal planes scanning of weight-bearing area. (**C**) Three-dimensional micro-CT images of medial subchondral bone compartment in coronal views. (**D**–**G**) The comparation between each group containing bone volume fraction (BV/TV; %), mean trabecular thickness (Tb.Th; /mm), average number of trabeculae (Tb.N; mm) and trabecular separation (Tb.Sp; mm), respectively. Statistical significance between the indicated groups is represented by *P<0.05, **P<0.01 or ***P<0.001 and the scale bar equals 600 μm in each group.

From representative two-dimensional micro-CT images (Figure 2A,B) and the corresponding three-dimensional modeling (Figure 2C), in both sagittal and coronal planes, the BV/TV parameter of the subchondral bone of the medial tibia in the DMM+veh group had clearly increased compared with the sham+veh group. Conversely, BV/TV values in the tramadol treatment groups were significantly lower than in both the DMM+veh and sham+veh groups. As shown in Figure 2D, compared with the sham+veh group (52.023 ± 3.214%), BV/TV measured in the DMM+veh group was demonstrably greater (64.703 ± 5.253%, *P*=0.0055). In addition, the BV/TV in the T10 (43.383 ± 2.459%), T20 (35.362 ± 3.067%) and T40 groups (36.102 ± 6.008%) were significantly less, *P*-values equaling 0.0722, <0.001 and <0.001, respectively. Thus, compared with the DMM+veh group, BV/TV in the DMM+T10, DMM+T20 and DMM+T40 groups were all lower, differences that were statistically significant (*P*<0.001). As shown in Figure 2E–G, compared with the sham+veh group, the Tb.Th in the DMM+veh group was higher (*P*<0.05) but less in the DMM+T20 group (*P*<0.005). Additionally, Tb.N decreased gradually in the DMM+T10, DMM+T20 and DMM+T40 groups, but the differences were not statistically significant. Furthermore, Tb.Sp increased gradually in the DMM+T10, DMM+T20 and DMM+T40 groups, differences that were statistically significant compared with the DMM+veh group (*P*<0.05). The value of Tb.Sp in the treatment groups had no significantly difference compared with that of the sham+veh group.

### Negative changes to elastic moduli measured by AFM as a result of using tramadol

**Figure 3:**
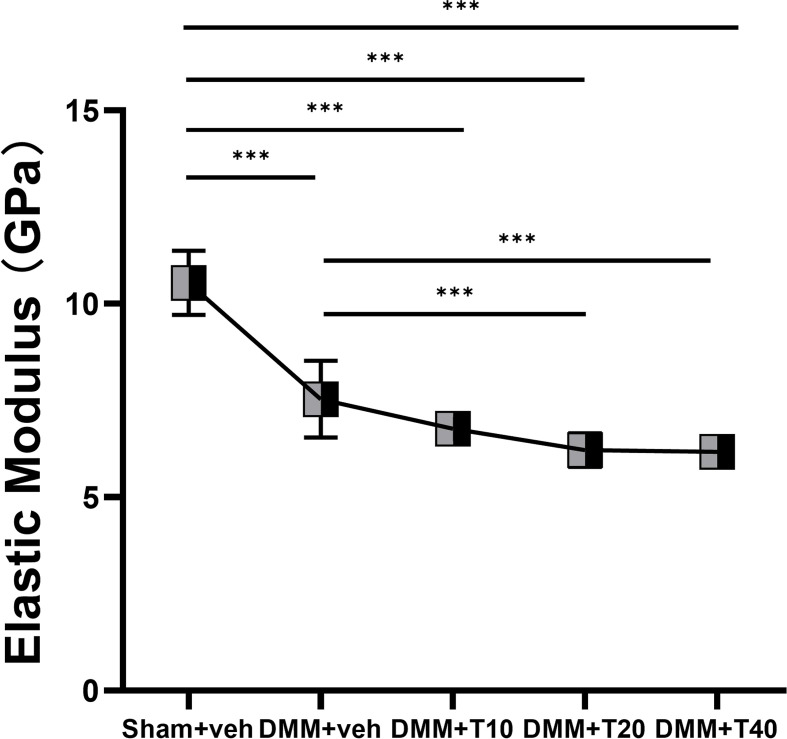
The analysis of AFM Quantitative analysis of the elasticity modulus of medial subchondral bone. Differences among groups are shown as ***P<0.001.

Changes in the elastic moduli of the subchondral bone are shown in Table 1 and Figure 3. The elastic moduli in the sham+veh group were highest at 10.541 ± 0.830 Gpa. In the DMM+veh group, the elastic moduli decreased to 7.53 ± 0.993 Gpa (*P*<0.001). In the three groups of OA treated with tramadol, the elastic moduli were 6.767 ± 0.258 Gpa, 6.220 ± 0.435 Gpa and 6.172 ± 0.366 Gpa in the DMM+T10, DMM+T20 and DMM+T40 groups, respectively. Therefore, compared with the sham+veh group, elastic moduli decreased with increasing tramadol concentration (*P*<0.001), although among the three tramadol groups there was no significant difference (*P*>0.05).

**Table 1:** The different points of subchondral bone in LBR of each sample were detected by probe (GPa)

Groups (*n*=5)	Sham+Veh	DMM+Veh	DMM+T10	DMM+T20	DMM+T40
Two points detected in each sample	10.468	7.003	6.566	5.601	6.512
	9.813	6.513	7.024	6.939	6.105
	9.845	6.735	7.099	6.066	5.933
	10.59	7.47	7.01	6.113	6.603
	12.016	9.068	6.651	6.482	5.793
	9.392	6.419	6.564	6.35	5.61
	11.06	8.636	6.345	5.948	6.439
	10.686	7.848	6.829	6.272	6.609
	11.571	8.786	7.002	5.664	5.837
	9.965	6.872	6.584	6.763	6.274

### Degeneration of cartilage evaluated by HE and SO/FG staining

**Figure 4:**
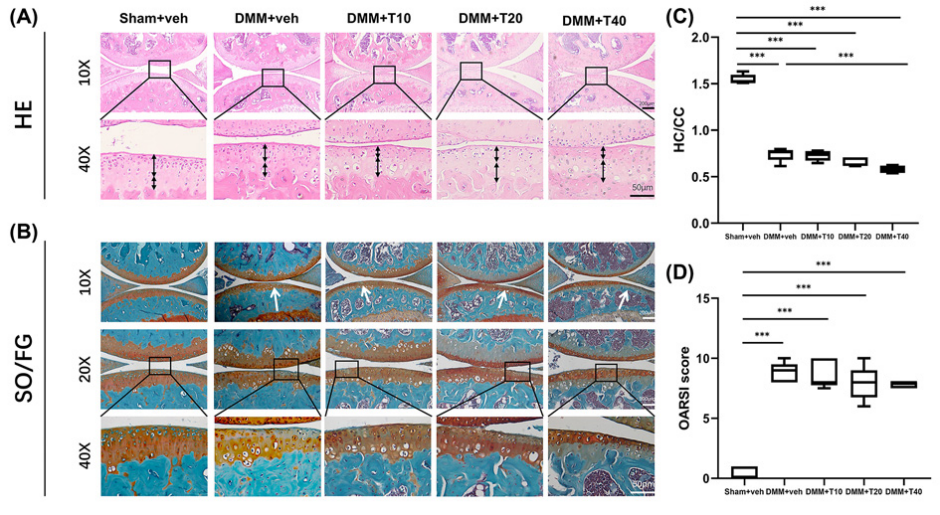
The analysis of cartilage by histopathological staining (HE and SO/FG) (**A,B**) Paraffin staining including HE and SO/FG staining of weight-bearing area of knee joints in mice. (**C**) Ratio of HC to CC in HE staining in groups. (**D**) OARSI scores of SO/FG staining. Statistically significant differences are indicated by where ***P<0.001 between the indicated groups. Scale bars in (A; above and bottom) were 100 and 50 μm, and in (B; above, middle and bottom) were 200, 100 and 50 μm, respectively.

From analysis of HE staining (Figure 4C), the ratio of HC to CC in the sham+veh group was 1.547 ± 0.0512, indicating that the HC in the sham+veh group was the thickest. In the DMM+veh group, the HC/CC value was 0.740 ± 0.0729 (*P*<0.001). That is, the HC layer had become thinner with tide lines moving higher which was obvious to be noticed in Figure 4A. In the treatment groups, HC/CC values were lower than in the DMM+veh group (0.727 ± 0.0551, 0.655 ± 0.0482, 0.580 ± 0.0395). Values in the DMM+T10 and DMM+T20 groups were not significantly different from that of the DMM group whereas values in DMM+T40 group were significantly lower than in the DMM+veh group.

SO/FG staining revealed that there was continuity of cartilage in the sham+veh group, preserved intact with no loss of proteoglycan (Figure 4B). Conversely, a rough surface was observed on the cartilage in the DMM+veh, DMM+T10, DMM+T20 and DMM+T40 groups with evidence of its continuous destruction and loss of proteoglycan from the superficial layer. Using OARSI scores (Figure 4D), the sham+veh group scored 0.400 ± 0.548 which was significantly lower than that of the DMM+veh group (8.800 ± 0.837) (*P*<0.001). Additionally, the scores of the DMM+T10, DMM+T20 and DMM+T40 groups (8.700 ± 1.204, 7.900 ± 1.432, 7.800 ± 0.274) were significantly higher than that of the sham+veh group (*P*<0.05) but not significantly different than the DMM+veh group.

### Synovitis scores from HE staining

**Figure 5:**
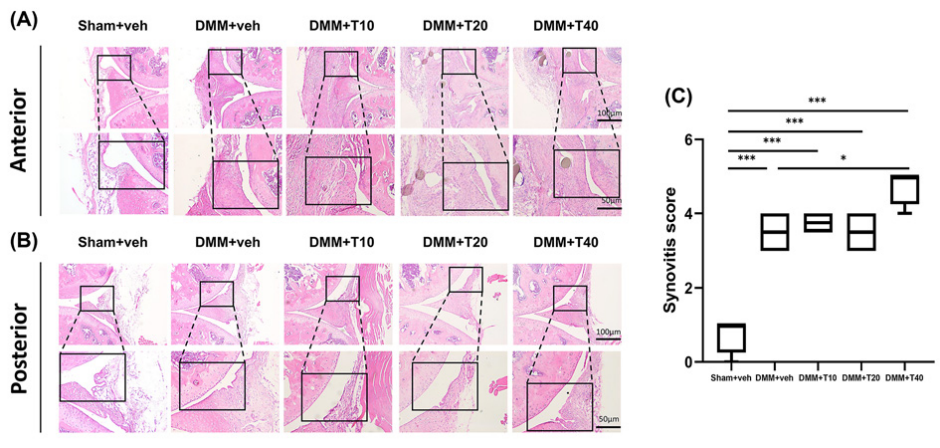
The analysis of synovium by histopathological staining (**A,B**) Representative pictures of synovial stained by HE in each group both in anterior and posterior sides of knee joints. (**C**) Synovial scores for synovial inflammation on mice. Statistically significant differences are indicated by where *P<0.05 or ***P<0.001 between the indicated groups. The scale bars equaling different lengths were shown above.

HE staining, which was conducted to observe the pathological changes in synovial tissues, demonstrated that sham mice exhibited no synovial hyperplasia and no inflammatory cell infiltration. In contrast, the groups treated by tramadol with different dosages had remarkable thickening of synovial lining cell layers, increasing density of the cells in the synovial stroma, synovial hyperplasia and more inflammatory cell infiltration, so did the DMM+veh group (Figure 5A,B). Therefore, it is obviously observed from the HE staining figures that the synovitis score of the sham+veh group was lower than those of all other groups (*P*<0.001). Furthermore, compared with the DMM+veh group, the synovitis score was unaltered in the DMM+T10 and DMM+T20 groups but slightly higher in the DMM+T40 group (*P*<0.05) (Figure 5C).

## Discussion

In the early stages of OA, it was found in the present study that the mass of subchondral bone in the knee joints of mice decreased in decompensation with a fall in elastic modulus that was more severe than that of the OA model when treated with tramadol. That is to say, use of tramadol in early OA appears to accelerate the decline of bone mass and elastic modulus of the subchondral bone, resulting in a negative prognosis for OA.

OA is among the most common degenerative joint diseases. However, its pathological mechanism remains unclear. The iconic pathogenesis of OA involves changes to joint microstructure and mechanical properties, as demonstrated by multiple research studies [27]. It is well known that in the normal knee joint, the spatial arrangement and biochemical characteristics of the subarticular bone and the internal bone trabeculae play an important role in supporting the articular surface in order to maintain its integrity and mechanical stability [28–31]. A change in microstructure and mechanical properties of the subchondral bone therefore play a critical role in maintaining the surface of the articular cartilage and even in OA. Clinically, physicians often prescribe drugs with different mechanisms such as tramadol, either alone or in combination with NSAIDs to relieve breakthrough pain. The combination of tramadol with NSAIDs as analgesic drugs in OA has a synergistic effect and allows a reduction in the dosage of NSAIDs [32,33]. Therefore, weak opioids, such as tramadol, have become a first-line drug for OA in terms of analgesia recommended by American College of Rheumatology [4,11]. The abuse of opioids is getting more widespread due to the strong analgesic effect of opioids, which leads to an increasing opioids prescriptions in patients with early OA [34,35]. However, the effect of weak opioids on the microstructure and mechanical properties of subchondral bone has not so far been reported. In the present study, we aimed to reveal the effects of tramadol in early OA, and to provide guidance for clinical treatment.

In the present study, OA was induced by DMM. In the early stages of OA (4 weeks after DMM), HE staining demonstrated fewer vacuoles, and so cells, in the cartilage and subchondral bone. Moreover, loss of proteoglycan and vertical fissures were observed in the medial tibia by SO/FG staining. These changes in the cartilage and subchondral bone are consistent with previous studies [19,36,37].

Bone mass altered and the quantity of sclerotin under the articular cartilage changed as OA progressed, depending on the intervention. From visual observation of the knee joints in the present study, the joint surface and attached chondrocytes were severely damaged to varying degrees after DMM surgery, destroying the stability of the knee joint. All visible changes were consistent with progression of OA, such as tissue section staining. From the microscopic images using SEM, the knee joints exhibited lacunae of varying depths after successful induction of OA, including samples from the DMM+veh and treatment groups, the number and sizes on the surface of the subchondral bone of which were characteristic of the bone destruction known to occur in the pathological progression of OA. In addition, a number of cracks on the surface of subchondral bone were observed, numerous and diffused in the DMM+veh and treatment groups more than that in sham+veh group. We found that chondrocytes attached to the bone surface degenerated to result in increased destruction of the subchondral bone during progression of OA. The results demonstrated that in the early stages of OA, the subchondral bone mass of the knee joint increased to a certain extent in order to adapt to the functional decompensation caused by the loss of joint stability [38–40]. However, in the mice treated with tramadol, the subchondral bone was less satisfactory than the sham+veh and DMM+veh groups, both in terms of bone mass and degeneration of sclerotin. This was due to the abnormal proliferation of osteoclasts and more activated function of bone resorption in the subchondral bone of the treatment groups. It could be inferred from these observations that treatment with tramadol in the early period of OA would abnormally accelerate the loss of bone mass and destruction of the subchondral bone.

Rather than changes in the bone mass, the effects of alterations to the mechanical properties of the subchondral bone are more intuitive. The elastic modulus measures the mechanical properties of a material on the micron scale, evaluated using a formula that explores the relationship of the force–time curve. In general, the elastic modulus represents the capability of a substance to resist deformation [41]. In the subchondral bone, this can extend to its ability to prevent fracture. During the progression of OA, in addition to changes in the microstructure, the mechanical properties also provided a different response to this lesion. According to the results of AFM analysis, the elastic modulus of the subchondral bone in the DMM+veh group was lower than in the sham+veh group, indicating that in the model of early OA, the degree of stiffness in the subchondral bone decreased due to pathological injury from OA and the ability to resist deformation and fracture inevitably diminished. Interestingly in mice treated with tramadol, the elastic modulus of their subchondral bone was lowest, and bone sclerosis, resistance to deformation and fracture of the subchondral bone were naturally inferior to joints from the DMM+veh group, let alone the sham+veh group. The AFM results demonstrated that the elastic modulus of the subchondral bone of the tibial plateau in the DMM+veh group was lower than that of the sham+veh group. Nevertheless, micro-CT suggested that there was mild sclerosis of the subchondral bone in the DMM+veh group. The possible reason for this might be that the bone exhibited characteristics of low levels of matrix mineralization, even if its density had increased. In the treatment groups, the elastic modulus decreased significantly compared with sham+veh and DMM+veh groups. In addition, loss of bone mass increased, indicating that the mechanical properties of the subchondral bone were more seriously affected.

The abnormal turnover and metabolism of the subchondral bone affects the progression of OA and is closely related to the destruction of cartilage. A number of published reports have observed that tramadol, combined with μ opioid receptors, inhibit osteocalcin synthesis in osteoblasts that interferes with bone metabolism [42]. In addition, there is relevance to bone metabolic changes and bone healing in tramadol which can inhibit the reuptake of serotonin [18,43]. In the early stages of OA in the DMM+veh group, the bone mass of the subchondral bone increased significantly, indicating increased bone turnover and metabolism. However, the decrease in mineral density and bone mass, the increase in bone cysts and the loose arrangement of trabecular bone in the subchondral tissue was apparent in the tramadol groups. These observations were significantly different from those in the sham+veh and DMM+veh groups. This phenomenon suggests that use of tramadol interfered with the remodeling of the subchondral bone resulting in an increased risk of osteoporotic changes and fragility fractures. According to the results of tissue staining, the surface of the cartilage of the DMM and treatment groups produced similar degeneration of thickness in the CC and HC compared with the sham+veh group. From the HE staining, it was apparent that the thickness of the HC on the surface of the joints in the DMM group was thinner than in the sham+veh group, and its thickness in the tramadol groups was thinnest. However, there was no significant difference in the morphology of the CC between the DMM and T groups. Based on the HC/CC ratio values in HE and the SO/FG OARSI scores, which grade the severity of cartilage injury, there was a degree of degeneration of the cartilage in the DMM and treatment groups, although the difference was not statistically significant. In addition, the quantity of glycogen lost from the cartilage surface and number of vertical fissures were similar in each case. The degree of inflammation caused by OA was measured by the number of synovial lining cell layers, synovial hyperplasia and inflammatory infiltration normally, and then quantified by the corresponding synovial scores [44]. The pathological situation, as reflected in the synovitis scores, was similar in the tramadol groups to that in the DMM+veh group, although more serious than in the sham+veh group. This suggests that administration of tramadol in the early stages of OA provided protection to the cartilage and synovium. Conversely, the cartilage and synovium would likely be aggravated by progression of OA on account of the degeneration of the microstructure and mechanical properties.

Considering the SEM, CT and AFM data, abnormal transformation of the subchondral bone contributed to a compensatory increase in bone mass and decrease in bone elastic modulus in early OA induced by DMM. On the other hand, articular cartilage was to a certain extent worn due to mechanical instability and the hypermetabolism of bone. In mice treated with tramadol in the early stage of OA, the subchondral bone was abnormally active in terms of bone metabolism, causing a decompensated decrease in bone mass and decrease in elastic modulus. Many indicators demonstrate that administration of tramadol in the early stages of OA had a negative effect on the microstructure and mechanical properties of subchondral bone. These factors could result in the loss of subchondral bone mass, disorder of trabecular structure and weakening of the ability of bone to resist deformation.

For treatment of early OA, attention should be focused on the remodeling of subchondral bone, loss of bone mass and reduction in elastic modulus in order to prevent osteoporosis. Therefore, in the early analgesic treatment of knee OA with tramadol, the effect of treatment on subchondral bone should be considered and appropriate measures carefully selected to avoid its interference on subchondral bone metabolism.

## Perspectives

- Tramadol is currently playing an increasingly important role in the treatment of OA. The purpose of the present study was to explore the effect of tramadol on the microstructure and mechanical properties of subchondral bone.
- In the current study, our results demonstrated that the administration of tramadol in the treatment of OA could accelerate the loss of bone mass, aggravate the destruction of its microstructure and lead to deterioration in mechanical properties of subchondral bone.
- Our results suggest that for the treatment of early OA, attention should be focused on subchondral bone in order to prevent OA. This means that tramadol may require additional adjuvant drugs in order to protect the subchondral bone.

## Abbreviations

AFM: atomic force microscopy
BV/TV: ratio of bone volume to total volume
CC: calcified cartilage
DMM: destabilization of the medial meniscus
DMM+veh: DMM+vehicle
HC: hyaline cartilage
HE: Hematoxylin and Eosin
micro-CT: micro-computed tomography
NSAID: non-steroidal anti-inflammatory drug
OA: osteoarthritis
OARSI: Osteoarthritis Research Society International
PBS: phosphate buffered saline
SEM: scanning electron microscopy
sham+veh: sham+vehicle
SO/FG: Safranin O-Fast Green
Tb.N: quantity of phalangeal trabeculae
Tb.Sp: sparsity of phalangeal trabeculae
Tb.Th: average thickness of phalangeal trabeculae
